# Spinal Cord Neuronal Network Formation in a 3D Printed Reinforced Matrix—A Model System to Study Disease Mechanisms

**DOI:** 10.1002/adhm.202100830

**Published:** 2021-08-05

**Authors:** Natalie Fischhaber, Jessica Faber, Ezgi Bakirci, Paul D. Dalton, Silvia Budday, Carmen Villmann, Natascha Schaefer

**Affiliations:** ^1^ Institute for Clinical Neurobiology University Hospital Würzburg Versbacherstr. 5 97078 Würzburg Germany; ^2^ Department of Mechanical Engineering Institute of Applied Mechanics Friedrich‐Alexander‐University Erlangen‐Nürnberg Egerlandstrasse 5 91058 Erlangen Germany; ^3^ Department of Functional Materials in Medicine and Dentistry and Bavarian Polymer Institute University Hospital Würzburg Pleicherwall 2 97070 Würzburg Germany; ^4^ Phil and Penny Knight Campus for Accelerating Scientific Impact University of Oregon 1505 Franklin Blvd. Eugene OR 97403 USA

**Keywords:** inhibitory synapses, mechanical testing, melt electrowriting, neuronal network maturation, nonlinear material behaviors, viscoelasticity

## Abstract

3D cell cultures allow a better mimicry of the biological and mechanical environment of cells in vivo compared to 2D cultures. However, 3D cell cultures have been challenging for ultrasoft tissues such as the brain. The present study uses a microfiber reinforcement approach combining mouse primary spinal cord neurons in Matrigel with melt electrowritten (MEW) frames. Within these 3D constructs, neuronal network development is followed for 21 days in vitro. To evaluate neuronal development in 3D constructs, the maturation of inhibitory glycinergic synapses is analyzed using protein expression, the complex mechanical properties by assessing nonlinearity, conditioning, and stress relaxation, and calcium imaging as readouts. Following adaptation to the 3D matrix‐frame, mature inhibitory synapse formation is faster than in 2D demonstrated by a steep increase in glycine receptor expression between days 3 and 10. The 3D expression pattern of marker proteins at the inhibitory synapse and the mechanical properties resemble the situation in native spinal cord tissue. Moreover, 3D spinal cord neuronal networks exhibit intensive neuronal activity after 14 days in culture. The spinal cord cell culture model using ultrasoft matrix reinforced by MEW fibers provides a promising tool to study and understand biomechanical mechanisms in health and disease.

## Graphical abstract

The studied spinal‐cord‐melt electrowritten‐frame‐hydrogel constructs reflect the highly nonlinear and time‐dependent behavior of ultrasoft nervous tissue. Offering this extracellular matrix surrounding allows maturation of the inhibitory synaptic network in a similar temporal resolution to native spinal cord tissue. Therefore, the model system resembles an excellent tool to study disease pathologies providing a better understanding of the underlying physiological processes.

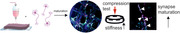

## 1 Introduction

Since the vaccine development of the 1940s and 1950s,^[^ ^1^ ^]^ scientists have been successfully culturing mammalian cells in laboratories in vitro in a 2D environment. Today, cell culture is a well‐established technique that has allowed researchers countless insights into the mechanisms of physiological processes in the human body in health and disease conditions. However, one limitation of 2D cell‐culture systems is that they do not fully reflect the physiological conditions existing inside the human body, as most cells grow in vivo within a 3D environment.^[^ ^2^, ^3^, ^4^ ^]^ In particular, cells that grow on the surface of a typical 2D Petri dish environment experience a supra‐physiological stiffness.^[^ ^4^ ^]^ Furthermore, cells are limited to growing in a monolayer^[^ ^4^ ^]^ and interact to a greater extent with the substrate than with other cells, reducing cell to cell communication.^[^ ^4^, ^5^ ^]^ These limits of 2D cell culturing have led to the use of 3D cell cultures driven by tissue engineering approaches.^[^ ^6^ ^]^ While the initial focus of tissue engineering was to construct whole tissues or organs in vitro prior to the repair of failing tissue inside the human body,^[^ ^7^ ^]^ 3D cell cultures provide methods to better understand physiological mechanisms as they occur in vivo.

Cells in the central nervous system (CNS) sense and respond to their mechanical environment,^[^ ^8^, ^9^, ^10^ ^]^ which is critical for tissue maturation and plays an important role during disease, injury, and regeneration.^[^ ^11^ ^]^ The rigid 2D cell culture substrates contrast with native CNS tissue which exhibits an ultrasoft, highly nonlinear, and time‐dependent material behavior.^[^ ^12^ ^]^ Therefore, when aiming to identify CNS mimicking materials for tissue engineering and cell culturing, it is necessary to not only consider a single stiffness value of the substrate representative for small strains and a fixed strain rate. In addition, mechanical analyses on the composites containing both cells and matrix are required.^[^ ^13^, ^14^ ^]^

Here we print 3D small diameter poly(*ε*‐caprolactone) (PCL) microfiber frames, and use them to mechanically support weak Matrigel formulations, a matrix to mimic the CNS extracellular matrix (ECM).^[^ ^3^ ^]^ The small diameter fibres produced by melt electrowriting (MEW)^[^ ^15^ ^]^ not only provide mechanical reinforcement but topographical cues for neurite outgrowth.^[^ ^16^ ^]^ In addition, the volume occupied by such reinforcing frames remains less than 10 vol%, so that the majority of space is occupied by the matrix. Microfibers have been shown to substantially reinforce hydrogels including weak formulations of Matrigel so that handling of the composite is possible.^[^ ^17^ ^]^

As a neuronal model, we use a primary spinal cord culture. In the nerve‐muscle circuit, motoneurons of the spinal cord, once excited, fire action potentials toward the neuromuscular endplate, finally leading to muscle contraction. In contrast, Renshaw cells or other inhibitory neurons of the spinal cord balance the firing rate of the motoneurons by hyperpolarization thereby controlling the contraction of the muscles. This feedback control is enabled by inhibitory neurotransmission via glycine receptors (GlyR) present in motoneuron membranes. Disturbances in inhibitory signal transduction processes are associated with different forms of neurological disorders, such as hyperekplexia (OMIM 14 900, startle disease, stiff baby syndrome), stiff person syndrome, epilepsy, pain sensitization, autism, and agoraphobia.^[^ ^18^, ^19^, ^20^, ^21^, ^22^ ^]^ The underlying molecular and cellular mechanisms in those diseases are not fully understood yet, making a 3D cell culture system a suitable tool to provide better understanding of the disease pathologies.

GlyR subunit expression is developmentally regulated. Whereas before birth the *α*2 subunit is the predominant subunit, in the adult spinal cord, GlyRs mainly consist of *α*1/3‐subunits and *β*‐subunits in a 3*α*:2*β* stoichiometry.^[^ ^23^, ^24^, ^25^ ^]^ *α*1‐mRNA expression has been identified around **e** mbryonic day 14 (E14) in rat spinal cord and expression increased until **p** ost‐natal day 15 (P15).^[^ ^26^ ^]^ The *α*1 protein has been detected earliest following day 7 after birth in mice.^[^ ^27^ ^]^ Surface expression of the *α*1*β*‐ heteromeric receptor complexes is a prerequisite of a functional inhibitory synapse (Figure S1, Supporting Information). However, a functional inhibitory synapse requires the presence of other essential presynaptic or postsynaptic interacting proteins. At presynaptic sites, the vesicular inhibitory amino acid transporter (VIAAT = VGAT) plays a crucial role for the transport of the two inhibitory neurotransmitters GABA and glycine from the cytoplasm to synaptic vesicles.^[^ ^28^ ^]^ At the postsynaptic site, GlyRs are anchored by the scaffold protein gephyrin at synaptic sites.^[^ ^29^ ^]^

In this study, we investigate the maturation of inhibitory synapses in a 3D environment using quantitative immunocytochemical and protein biochemical approaches as well as calcium imaging. The biological part is complemented by large‐strain mechanical analyses to assess the tissue mimicking capabilities of the 3D model system. Our 3D spinal cord neuronal cell culture model provides a relatively easy to copy and easy to handle tool to study molecular pathomechanisms associated with spinal cord neurodegeneration.

## 2 Results

### 2.1 Sample Preparation and Mechanical Testing

**Figure 1:**
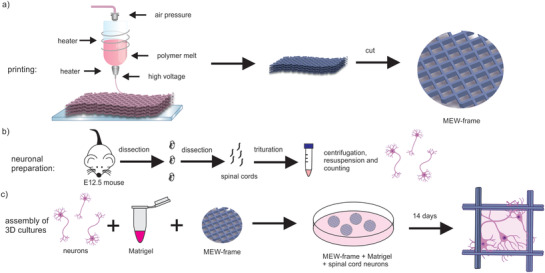
Schematics of 3D sample preparation. a) MEW‐printing process of the frame is shown. b) Isolation of neurons from E12.5 mouse embryos. c) Organization of 3D spinal cord culture by combining primary neurons, Matrigel as ECM and the MEW‐frame.

To experimentally build up an ultrasoft cell culture model resembling nervous tissue (brain or spinal cord) with a stiffness of only a few hundred Pa,^[^ ^30^ ^]^ we combined primary spinal cord neurons with Matrigel and a PCL‐based frame printed with MEW. Primary spinal cord neurons were prepared from E12.5 mouse embryos and Matrigel was used in low concentrations (4.5 mg mL^−1^) as a matrix. Combining neurons and 4.5 mg mL^−1^ Matrigel alone results in constructs that are unable to handle.^[^ ^17^ ^]^ Therefore, the PCL‐based frame (Video S1, Supporting Information) was used to reinforce the matrix and to enable handling of the whole composite.^[^ ^3^ ^]^ The suspension with 450 000 cells and Matrigel was pipetted on top of fabricated MEW‐frames and flooded with medium following gelation (**Figure** **1**).

**Figure 2:**
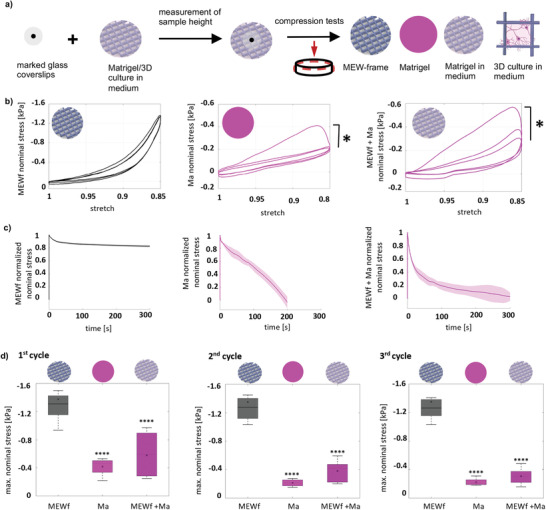
Schematic illustration of sample preparation and mechanical testing. a) A marked glass coverslip was used to determine the sample height and to define the undeformed (solid black lines) configuration. The deformed (dashed red lines) configuration describes the sample during compression in a rotational rheometer with axial motorization: MEW‐frame (MEWf), pure Matrigel (Ma), MEW‐frame‐Matrigel (MEWf + Ma) in medium. b) Representative preconditioning behavior in unconfined cyclic compression up to a maximum strain of 15% for MEW‐frame (*n* = 9), pure Matrigel (*n* = 5), and MEW‐frame‐Matrigel samples (*n* = 11), significance value *p < 0.05. c) Normalized stress relaxation behavior with corresponding standard deviations of MEW‐frame (*n* = 8), pure Matrigel (*n* = 5), and MEW‐frame‐Matrigel samples (*n* = 10). d) Maximum nominal stress (mean ± SD) during the first, second, and third loading cycles for all samples tested in unconfined cyclic compression, significance value   ****p < 0.0001. Significances were calculated using students *t‐*test.

To verify that the constructs can mimic the mechanical properties of native tissue, we performed mechanical analyses under large strains and different loading conditions (**Figure** **2**a).

The average response of the MEW‐frame, pure Matrigel, and MEW‐frame‐Matrigel samples was analyzed during three cycles of unconfined compression with a maximum strain of 15% (Figure 2b). While the MEW‐frame alone showed no clear differences between the individual loading cycles, both Matrigel and MEW‐frame‐Matrigel samples exhibited significant preconditioning effects with a stiffer response during the first loading cycle than during subsequent cycles (Table S1, Supporting Information). Interestingly, this mechanical response of the MEW‐frame‐Matrigel composites resembled the conditioning behavior found in native brain tissue.^[^ ^12^ ^]^ In addition, MEW‐frame‐Matrigel samples show a more pronounced hysteresis—defined as the enclosed area between the loading and unloading curve—than pure Matrigel and the MEW‐frame. This hysteresis quantifies the energy dissipation during cyclic compression and indicates higher viscous effects for Matrigel and the MEW‐frame‐Matrigel samples than for the more elastic MEW‐frame. While determining the averaged stress relaxation response with standard deviations, the samples were held at a constant strain of 15% compression for 300 s (Figure 2c). A slight decrease in stresses for the MEW‐frame with an equilibrium value of 82% of the initial stress confirms the rather elastic behavior. In contrast, the stresses for Matrigel have completely relaxed after 200 s. Besides handling advantages, the combination of MEW‐frame and Matrigel prevents the Matrigel from losing its form stability and drying out. The corresponding stress relaxation curve underlines the extreme time‐dependence of the MEW‐frame‐Matrigel samples with a stress relaxation up to 97% of the initial stress value within 300 s. Figure 2d summarizes the maximum nominal stresses for the MEW‐frame, pure Matrigel, and MEW‐frame‐Matrigel samples reached during all three loading cycles. For the first cycle, there are significant differences between the MEW‐frame and both the Matrigel (****p = 0.000 02) as well as MEW‐frame‐Matrigel (****p = 0.00 004) maximum stress values. For the second and third cycles, these differences are even more pronounced (see Table S1, Supporting Information, for *n* numbers, mean ± SEM, and *p*‐values). Interestingly, there was no significant difference in the maximum nominal stresses between the pure Matrigel and the MEW‐frame‐Matrigel composites.

### 2.2 Protein Expression of Inhibitory Synaptic Marker in Native Murine Spinal Cord

**Figure 3:**
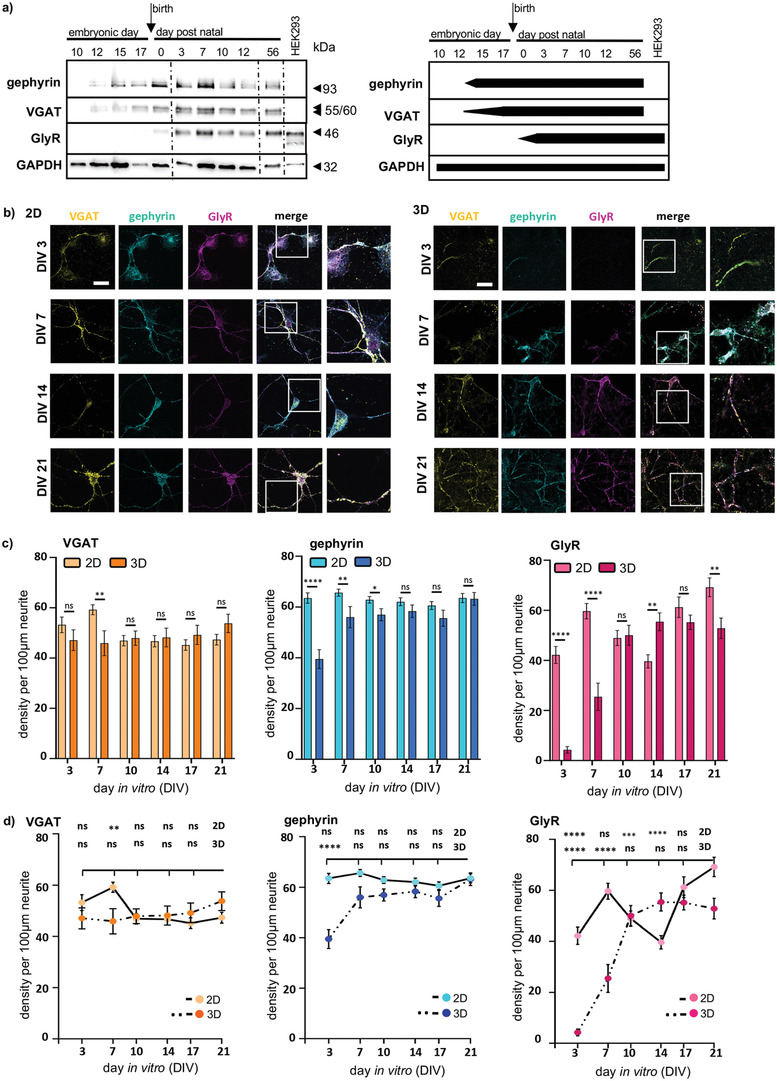
Expression of marker proteins at inhibitory synapses. a) Left: Western blot of mouse spinal cord membrane preparations from embryonic days: E10, E12, E15, E17, and postnatal days: P3, P7, P10, P12). GlyR*α*1 transfected HEK293 cells were used as positive control. Gephyrin (93 kDa), VGAT (55/60 kDa), GlyR (48 kDa), and GAPDH (32 kDa) were stained (black arrow heads). GAPDH was used as a loading control. Right: Schematic illustration of protein expression level. b) Immunocytochemical staining of 2D (left) and 3D (right) primary spinal cord cultures at DIVs 3, 7, 14, and 21. Gephyrin (cyan), GlyR (magenta), and VGAT (yellow) were stained (merge and magnifications, right two columns). Scale bar refers to 50 µm. c) Densities per 100 µm neurite of stained proteins (gephyrin cyan, 2D: *n* = 158–200, 3D: *n* = 47–80, GlyR magenta 2D: *n* = 81–100 3D: *n* = 17–40, and VGAT yellow, 2D: *n* = 79–100; 3D: *n* = 17–40) are depicted in 2D (lighter bar) compared to 3D (darker bar) at different days in culture. Significances were calculated using students *t‐*test. d) Line plots 2D and 3D with VGAT gephyrin and GlyR, densities per 100 µm neurite over time. Comparisons were analyzed by one‐way‐ANOVA.

Following mechanical testing as a prerequisite that our constructs resemble a similar environment as spinal cord or brain, we studied the protein expression level of inhibitory synaptic proteins in native spinal cord tissue. Gephyrin, VGAT, and GlyR expression were investigated using membrane preparations of mouse spinal cord tissue in samples before birth and at postnatal stages (E10, E12, and E17 and P0, P3, P7, P10, and P12). While gephyrin and VGAT expression starts already before birth between E10 and E12, the onset of the GlyR*α*1 expression is after birth (**Figure** **3**a). This expression pattern is in line with previous published data.^[^ ^28^, ^31^, ^32^ ^]^

### 2.3 Protein Expression in Manufactured 3D Versus 2D Spinal Cord Cultures

For analysis of the protein expression in 2D and 3D primary neuronal cultures, fluorescence densities of the marker proteins at inhibitory synapses were quantified at various time points (day in vitro = DIV) (Figure 3b). According to native spinal cord tissue, gephyrin, and VGAT should be present after neuronal seeding in cultures, whereas the inhibitory GlyR is assumed to be upregulated during the second week in culture reflecting postnatal stages. The presynaptic transporter VGAT displays a stable expression level in 2D and 3D with comparable densities. At DIV7 a significant difference between 2D and 3D is noted (DIV7: **p = 0.0037; see Table S2 for *n* numbers, mean ± SEM, and *p*‐values, Supporting Information) possibly reflecting a later expression onset in 3D following matrix adaptation. Like VGAT, the postsynaptic marker gephyrin is present in 2D and 3D cultures 3 days after seeding (see Table S2 for *n* numbers, mean ± SEM, and *p*‐values, Supporting Information). Although gephyrin onset is delayed in 3D MEW‐matrix composites compared to 2D (significant increase in 2D between DIV3‐10; DIV3 ****p < 0.0001, DIV7 **p = 0.0057, and DIV10 *p = 0.0217), it reached a similar expression level within the second week in culture (see Table S2 for *n* numbers, mean ± SEM, and *p*‐values, Supporting Information). The inhibitory GlyR was already observed at the first day of analysis (DIV 3) although with rather low values in 3D cultures. The onset of GlyR expression in a 2D culture was earlier compared to 3D (Figure 3c). Following adhesion to poly‐lysin coated cover slips in 2D, neurons start directly with expression of the GlyR (significant increases at DIV3 ****p < 0.0001, DIV7 ****p = 0.0000, DIV14 **p = 0.0011, and DIV21 **p = 0.0080) dissimilar to the in vivo situation (Figure 3a,b,d; see Table S2 for *n* numbers, mean ± SEM, and *p*‐values, Supporting Information). The 3D neuron‐matrix composite better reflects the expression data obtained from native spinal cord (Figure 3a,d).

### 2.4 Colocalization of Synaptic Marker in 3D Reflects Temporal Formation of Mature Synapses In Vivo

**Figure 4:**
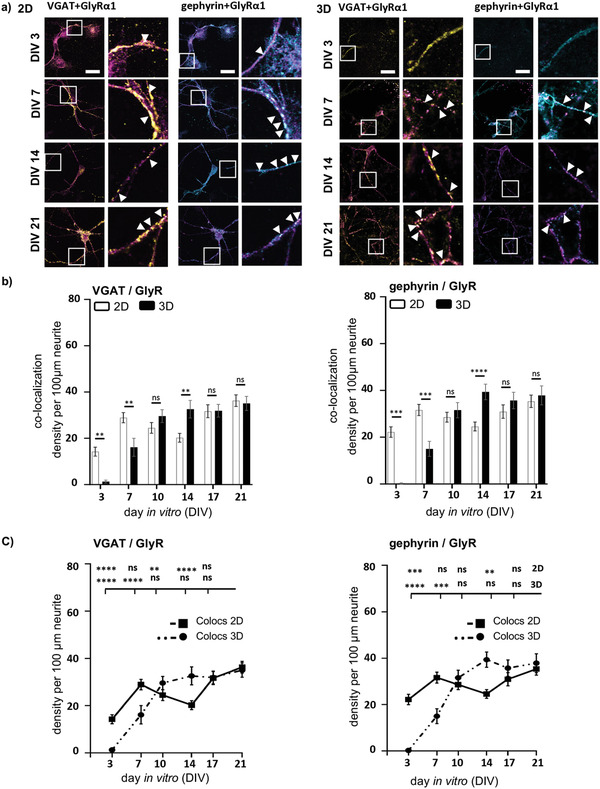
Postsynaptic protein colocalization. a) Colocalization analysis of 2D (left) and 3D (right) primary spinal cord cultures between DIV3‐21. Colocalization of VGAT (yellow) with GlyR (magenta) (left) and gephyrin (cyan) with GlyR (magenta) are shown. Right image per group displays a magnification, arrow heads point to colocalization spots. Scale bar refers to 50 µm. b) Density per 100 µm neurite of each colocalization (VGAT/GlyR 2D: *n* = 30–100, 3D: *n* = 17–40, and gephyrin/GlyR 2D: *n* = 80–100, 3D: *n* = 17–40) is shown with 2D (white bar) compared to 3D (black bar). Significances were calculated using students *t‐*test. c) Line plot of 2D and 3D VGAT/GlyR (left) and gephyrin/GlyR (right) densities per 100 µm neurite. *S*ignificances were calculated using one‐way‐ANOVA.

Besides total expression levels, colocalization of pre‐ and postsynaptic marker proteins is an indicator for a mature functional synapse. To investigate structurally mature inhibitory synapses, a colocalization analysis of VGAT and GlyR as well as gephyrin and GlyR was performed using a colocalization tool in ImageJ (**Figure** **4**a).

The colocalization profile of the marker proteins at the inhibitory synapses deliberates the expression pattern of the GlyR. Due to early expression of the GlyR in 2D cultures, receptor proteins colocalize with the presynaptic VGAT and the postsynaptic gephyrin already at DIV3 (Figure 4a–c; see Table S3 for *n* numbers, mean ± SEM, and *p*‐values, Supporting Information). In 3D cultures, colocalization of the GlyR with VGAT and gephyrin is continuously increasing until DIV10, where it reaches a plateau (VGAT 3D: DIV21 vs DIV3: ****p < 0.0001; DIV21 vs DIV7: ***p = 0.003) (Figure 4c; see Table S3 for *n* numbers, mean ± SEM, and *p*‐values, Supporting Information). GlyR gephyrin colocalization in 3D is highest at DIV14 and stays constant until DIV 21 (see Table S3 for *n* numbers, mean ± SEM, and *p*‐values, Supporting Information). In summary, the maturation of the spinal cord neuronal network in a 3D neuron‐matrix composite reflects the in vivo situation requiring 2–3 weeks after birth to form structurally and functionally adult inhibitory synapses.

### 2.5 GlyR*α*1 Surface Expression in Manufactured 3D Versus 2D Spinal Cord Cultures

**Figure 5:**
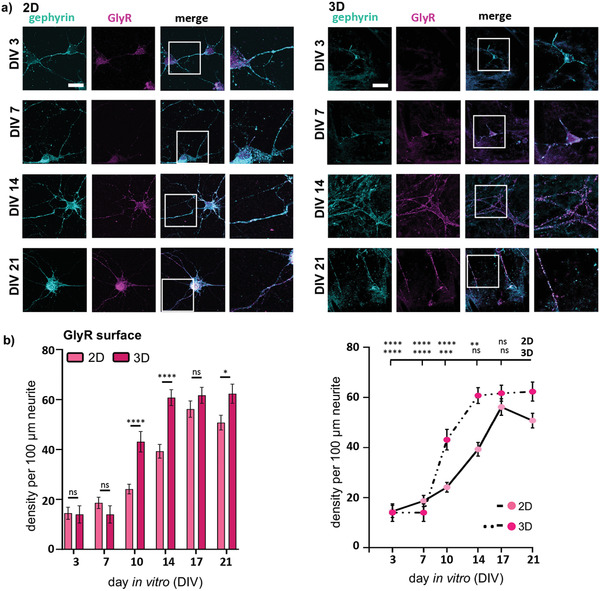
Surface expression of GlyR in 2D compared to 3D. a) Immunocytochemical staining of 2D (left) and 3D (right) primary spinal cord cultures between DIV3‐21. Whole cell gephyrin (cyan) and surface GlyR*α*1 (magenta) were stained (merge and magnification is shown in right 2 columns). Scale bar refers to 50 µm. b) Left: Densities per 100 µm neurite for surface GlyR (magenta) is shown in 2D (lighter bar) compared to 3D (darker bar) (2D: *n* = 78–100, 3D: *n* = 30–40) at different days in culture. Significances were calculated using students *t‐*test. Right: Line plot of 2D and 3D surface GlyR (magenta) densities per 100 µm neurite comparing each day in culture with DIV21 using one‐way‐ANOVA is depicted.

During maturation of adult inhibitory synapses, GlyR expression switches from *α*2 to *α*1*β* and *α*3*β* receptors.^[^ ^23^, ^24^, ^25^ ^]^ Therefore, the surface expression of the *α*1 subunit is a better indicator for adult synaptic GlyRs expressed in the outer membrane of neurons. During the trafficking process to the neuronal membrane, GlyR *α* and *β* subunits are cotransported in a complex together with gephyrin and the motor protein kinesin to synaptic sites.^[^ ^33^ ^]^ Thus, we compared the surface expression of the inhibitory GlyR*α*1 in 2D versus 3D (**Figure** **5**a).

Interestingly, in both 2D and 3D cultures surface GlyR expression levels start with similar densities and increase over time (Figure 5a,b). Hence, the observed early expression of the GlyR in 2D when considering the whole cell receptor expression point to an earlier onset of intracellular GlyR expression rather than surface expression (Figure 3). In 3D, the surface expressed GlyR is significantly increased compared to 2D at DIVs 10 (****p < 0.0001), 14 (****p < 0.0001), and 21 (*p = 0.0207) (Figure 5b, see Table S4 for *n* numbers, mean ± SEM, and *p*‐values, Supporting Information). Moreover, following an adaption to the 3D surrounding, the expression of the surface GlyR showed a steeper increase between DIV7 and 10 compared to 2D reaching the maximum at DIV 14, whereas in 2D GlyR expression is highest at DIV17 but slightly decreased until DIV21.

### 2.6 Colocalization of GlyR*α*1 Surface and Intracellular Gephyrin Proteins in Manufactured 3D Versus 2D Spinal Cord Cultures

**Figure 6:**
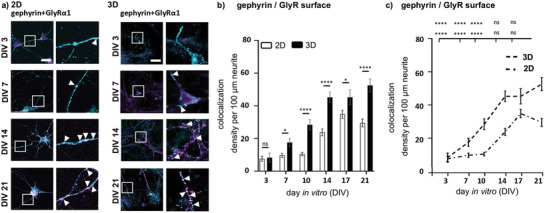
Colocalization analysis of surface GlyR with gephyrin in 2D versus 3D. a) Colocalization of immunocytochemical staining of left 2D and right 3D primary spinal cord cultures at DIV3‐21. Colocalization of whole cell gephyrin (cyan) with GlyR (magenta) are shown. Right picture per group displays a magnification, arrow heads mark colocalization spots. Scale bar refers to 50 µm. b) Densities per 100 µm neurite of gephyrin/GlyR colocalization is shown in 2D (white bar) compared to 3D (black bar) at different days in culture (2D: *n* = 78–100, 3D: *n* = 20–40). Significances were calculated using students *t‐*test. c) Line plot of 2D and 3D gephyrin/GlyR densities per 100 µm neurite, significances were calculated using one‐way‐ANOVA.

As previously mentioned, the intracellularly expressed protein gephyrin anchors the inhibitory GlyR at the inhibitory synapse. It can be assumed that colocalization of both surface GlyR expression and intracellular gephyrin expression counts for a functional inhibitory synapse.^[^ ^34^ ^]^ Colocalization analysis of surface GlyR with gepyhrin revealed an increase in colocalization of both proteins over time in both 2D and 3D spinal cord cultures (**Figure** **6**a–c). A significantly higher colocalization level of GlyR and gephyrin was observed in the 3D model system compared to the 2D monolayer approach at all days (DIV7: *p = 0.0125; DIV10: ****p < 0.0001; DIV14: ****p < 0.0001; DIV17: *p = 0.0324; DIV21: ****p < 0.0001) (Figure 6b; see Table S4 for *n* numbers, mean ± SEM, and *p*‐values, Supporting Information). In addition, colocalization in 2D dropped down after DIV17, while still increasing in 3D between DIV14 and 21. One might speculate that better nutrient supply, the 3D space or changes in biomechanical properties may underlie those observations.

### 2.7 Mechanical Testing and Functional Investigation of 3D Constructs Containing Neurons, Matrigel, and MEW‐Frame

**Figure 7:**
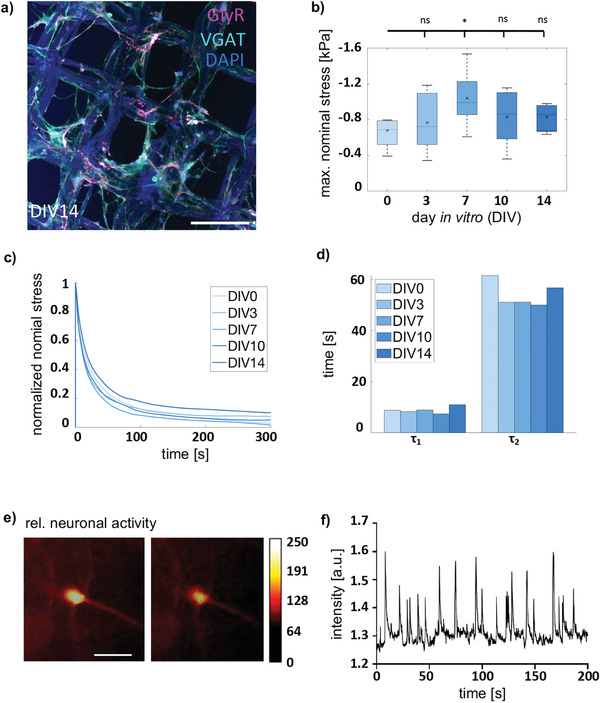
Mechanical testing of 3D primary spinal cord cultures at DIV0, 3, 7, 10, and 14. a) Immunocytochemical z‐stack of a DIV14 composite. Proteins marked are VGAT (cyan), GlyR (magenta), Nuclei (DAPI), MEW‐scaffold shows an autofluorescence in the DAPI‐channel. Scale car refers to 200 µm. b) Maximum nominal stresses (mean ± SD) during the first loading cycle in unconfined compression up to a maximum strain of 15% for 3D primary spinal cord cultures at DIV0, DIV3, DIV7, DIV10, and DIV14 (*n* = 10), significance value *p < 0.05. c) Normalized stress relaxation behavior for all 3D culture samples (*n* = 10). d) Time constants *τ*_1_ and *τ*_2_ obtained by fitting a two‐term Prony series to the stress relaxation curves in c). Significances were calculated using one‐way ANOVA followed by Tukey–Kramer tests for multiple comparisons. e) Calcium imaging of spinal cord neurons at DIV14. Same cell with high (left) and low (right) neuronal activity. Relative neuronal activity is shown by color code for intensity of the signals from dark brown (no activity) to white (high activity). Scale bar refers to 50 µm. f) Spontaneous action potential firing of a spinal cord neuron DIV 14 over a time course of 300 s.

The biomechanical properties of matrices have an impact on cell survival and maturation. Neuronal network formation required up to 3 weeks in culture. During this process, neurons form extensive dendritic networks and undergo cell–cell interactions by synapse formation (**Figure** **7**a). All these processes influence the biomechanical properties of the surrounding matrix. Therefore, 3D mature constructs were tested for changes in mechanical properties and compared to MEW‐frame‐Matrigel samples without cells. Figure 7b illustrates the average maximum stresses with standard deviations during the first cycle of compressive loading up to 15% strain for 3D primary spinal cord cultures at DIVs 0, 3, 7, 10, and 14. The stresses increase up to DIV7 (*p = 0.0258) followed by a slight decrease up to DIV14 (see Table S5 for *n* numbers, mean ± SEM, and *p*‐values, Supporting Information). In general, the highest stress values are measured for DIV7, DIV14, and DIV10 (in descending order). This increase in stiffness could be explained by the neural network maturation, which starts after DIV7 in 3D primary spinal cord cultures. These data are in line with previous results on cortical neurons^[^ ^17^ ^]^ and seem to be accompanied by a temporal gradient in the samples’ mechanical properties. In general, the addition of spinal cord neurons leads to an increase in stiffness of 17% (DIV0), 32% (DIV3), 79% (DIV7), and 44% (DIV10/14) compared to the MEW‐frame‐Matrigel samples without cells analyzed in Figure 2.

The stress relaxation behavior of 3D primary spinal cord cultures at DIV0, DIV3, DIV7, DIV10, and DIV14 exhibited pronounced viscoelastic effects, where 90–98% of the initial stress is relaxed within only 300 s (Figure 7c). All 3D samples showed a similar stress relaxation response, independent of the network maturation. Compared to the MEW‐frame‐Matrigel samples without cells (Figure 2c), there are no significant differences in the relaxation behavior, which indicates that the inclusion of 450 000 spinal cords cells per MEW‐frame does not affect the viscous properties of the samples. The stress relaxation behavior (Figure 7c) was further quantified using a multiterm Prony series^[^ ^12^, ^35^, ^36^ ^]^

(1) $$\sigma \;\left(t\right)=\sigma_{\infty }\;+\sum_{i\;=\;1}^{n}\left[\sigma_{i}-\sigma_{\infty }\right]\cdot e^{\left(-t/\tau_{i}\right)}$$

with *n* characteristic time constants *τ*_*i*_. The plateau stress *σ*_∞_ at *t* → ∞ is related to the storage modulus and characterizes the elastic response, whereas the difference between peak stress and plateau stress [*σ*_*i*_ − *σ*_∞_] is associated with the loss modulus and characterizes the viscoelastic response. Similar to previous studies on brain tissue,^[^ ^35^, ^36^, ^37^ ^]^ we found that a two‐term Prony series satisfactorily approximates the time‐dependent behavior of 3D cultures in medium. The corresponding time constants *τ*_1_ and *τ*_2_ for DIV0, DIV3, DIV7, DIV10, and DIV14 are summarized in Figure 7d. The fast time constant *τ*_1_ is similar for all 3D cultures, whereas the slow time constant *τ*_2_ shows slightly longer relaxation times for DIV0 (*τ*_1_= 7–11 s, *τ*_2_= 50–62 s). Figure 7c depicts this slower response of DIV0 by a more distinct decrease in nominal stress up to 300 s of stress relaxation.

To further investigate the functional maturation of the 3D neural network, calcium imaging was performed (Figure 7e,f). At DIV14 in 3D spinal cord cultures, neuronal activity was observed demonstrating that the mature inhibitory synapses resulted in functional network activity. Neurons in 2D monolayers at the same age exhibited only low Ca^2+^ signals upon activation (data not shown).

## 3 Discussion

The cellular environment in the CNS represents a particularly complex matrix with highly nonlinear and time‐dependent material behavior.^[^ ^38^ ^]^ A key challenge when conducting studies with neural tissue is to mimic the ultrasoft native CNS tissue with an elastic modulus of only a few hundred Pa.^[^ ^30^ ^]^ Previously, it was shown by neuronal differentiation and maturation approaches that 3D cell cultures of neurons require ultraweak matrices to enhance neuronal differentiation and maturation.^[^ ^39^ ^]^ Ultrasoft matrices alone, however, are unable to handle and take through the cell culture process or to allow transfer into an electrophysiological recording chamber. Therefore, reinforcement of the hydrogel is required and has been successfully demonstrated for cortical neurons and transfected cell lines.^[^ ^3^, ^17^ ^]^ The present study makes use of MEW‐reinforced Matrigel to study murine spinal cord neuronal maturation in a 3D model compared to a 2D monolayer approach.

Neurons of the spinal cord once excited transmit action potential to the neuromuscular endplate. Following further signal processing muscle contraction is enabled. Other neuronal populations in the spinal cord, e.g., interneurons control these processes by feedback inhibition, which is facilitated by inhibitory glycine receptors.^[^ ^40^ ^]^ Disturbances in spinal cord signal processing is associated with different neurological diseases such as stiff baby syndrome, stiff person syndrome, epilepsy, autism, and panic disorders.^[^ ^18^, ^19^, ^20^, ^21^, ^22^ ^]^ The underlying molecular and cellular pathologies are not completely understood yet, forcing us to develop a 3D model system that allows studying disease mechanisms.

Our 3D constructs consist of primary spinal cord neurons (taken at embryonic stage from mice) embedded in Matrigel reinforced by MEW PCL microfibers printed in a box‐like scaffold.^[^ ^3^ ^]^ Matrigel is derived from the Engelbreth‐Holm‐Swarm mouse sarcoma and hence includes ECM proteins, such as laminin, collagen, and growth factors playing an important role in neuronal differentiation.^[^ ^41^ ^]^ Laminin is also commonly used for coating of glass slides or plastic dishes to study neuronal networks in a monolayer approach.^[^ ^42^ ^]^ Other options for coating are represented by poly‐ornithine and poly‐lysine used in this study.^[^ ^43^ ^]^

To allow cellular growth, the matrix‐MEW‐frame composite must mimic the mechanical properties of native tissue. The MEW‐frames alone showed higher maximum nominal stresses and less pronounced hysteresis than pure Matrigel. The material behavior of the frames is therefore mostly elastic, while pure Matrigel exhibits highly viscous effects. When combining the MEW‐frame and Matrigel, it becomes possible to resemble the highly nonlinear and time‐dependent behavior (characterized by hysteresis, conditioning, and stress relaxation) of ultrasoft nervous tissue, such as brain or spinal cord tissue.^[^ ^12^, ^44^, ^45^ ^]^

Spinal cord neuronal maturation in 3D MEW‐frame‐Matrigel composites reflects the in vivo situation. Maturation was determined by expression analysis of marker proteins required for functional inhibitory synapse formation. The presynaptic marker VGAT as well as the postsynaptic marker gephyrin were present already at DIV3 in 2D and 3D cultures and were expressed stably during the time‐course of analysis.^[^ ^46^ ^]^ Day3 in our primary neuronal culture reflects the embryonic stage around E16 where a clear protein signal was detectable in spinal cord tissue preparations. During CNS development, the expression start of VGAT is supposed to be by E17.5 in the mouse spinal cord.^[^ ^47^ ^]^ For gephyrin first expression at the mRNA level have been reported at day E14.^[^ ^48^ ^]^ For the GlyR, in tissue and 3D cultures the GlyR*α*1 was very low in expression at the protein level. Previously, we could show that the GlyR*α*1‐subunit can be detected in spinal cord tissue at postnatal stage P7.^[^ ^27^ ^]^ A postnatal stage P7 is mirrored by the second week in culture where the GlyR expression reaches almost the maximum of expression in 3D cultures.

2D constructs gave rise to detectable GlyRs after 3 days in culture arguing for initial upregulation in a monolayer approach. This phenomenon was observed previously.^[^ ^46^ ^]^ Here, spinal cords were dissected from rats at the embryonic stage E14. Fluorescence intensity was maximal at DIV4‐5 and decreased after DIV7. This effect was however only observed when whole cell GlyR expression was determined. GlyR surface expression and thus receptors at fully functional synaptic sites do not resemble this expression pattern. Expression of the mature *α*1 containing heteromeric receptors incorporated in motoneurons membranes was delayed in 2D compared to 3D reaching the maximum level at DIV17 in 2D compared to DIV14 in 3D. A slight decline of receptor expression was visible in both cell cultures within the third week in culture when maturation is almost complete.

Further evidence about the initial upregulation followed by stable protein expression and a possible protein expression decline is provided from the literature. Due to maturation processes at the synapse, synapses undergo synaptic reconstruction, synaptic strengthening of synapses that have received high neural input and pruning of redundant synapses.^[^ ^49^, ^50^, ^51^ ^]^ Colocalization of the GlyR*α*1 with gephyrin provides a further hint for structurally and functionally mature inhibitory synapses. However, 3D cultures exhibited twice as many colocalized gephyrin/GlyR clusters compared to 2D cultures most probably due to the better mimicking of the native ECM surrounding. These data were accompanied by estimation of a fully functional 3D spinal cord neuronal network after 14 days in culture, while neurons in 2D exhibited much less neuronal firing.

Our mechanical analyses of the 3D constructs including primary spinal cord neurons demonstrated that the samples display maximum stresses of 0.7–1.0 kPa during cyclic unconfined compression and viscoelastic effects, i.e., a pronounced hysteresis, conditioning, and stress relaxation. Moreover, the inclusion of spinal cord cells and their maturity levels in 3D primary spinal cord cultures affected the mechanical response of the constructs. The stresses during the first loading cycle in compression increased by 17% on day 0 up to 79% on day *7* in vitro compared to composites without cells. After day 7 in vitro the stresses slightly decreased again. We attribute the significant increase on DIV7 to the start of the neural network maturation after 7 days in vitro.^[^ ^17^ ^]^

To quantify the complex time‐dependent nature of 3D primary spinal cord cultures, we adopted a two‐term Prony series to identify characteristic time constants.^[^ ^35^, ^36^ ^]^ We found that two time‐constants with values of *τ*_1_= 7–11 s and *τ*_2_= 50–62 s were sufficient to approximate the stress relaxation response of the constructs. The short time scale *τ*_1_ is slightly higher and the long‐time scale slightly lower than values previously reported for brain tissue (brain tissue: *τ*_1_= 2–3 s and *τ*_2_= 67–120 s,^[^ ^52^ ^]^ but they lie within the same range. In conclusion, 3D primary spinal cord cultures well resemble the highly nonlinear and time‐dependent behavior of ultrasoft nervous tissue.

## 4 Conclusion

In summary, our 3D MEW‐frame‐Matrigel spinal cord neuronal culture is a reliable and suitable test‐system to investigate in vivo processes in the CNS. The used MEW‐frame‐hydrogel reflects the highly nonlinear and time‐dependent behavior of ultrasoft nervous tissue offering a suited ECM surrounding that allows maturation of the inhibitory synaptic network in a similar temporal resolution to native spinal cord tissue. Hence, the system represents an excellent tool to study disease pathologies providing a better understanding of the underlying physiological processes.

## 5 Experimental Section

### MEW Reinforcement Frames

PCL (Corbion Inc, Netherlands, PURASORB PC12, Lot#1 412 000 249, 03/2015) frames were fabricated as previously described.^[^ ^53^ ^]^ MEW was carried out at a PCL heating temperature of 80 °C, an ambient temperature of 21 ± 2 °C and a humidity of 35 ± 10%. Frames were produced by direct‐writing an electrified jet across a collector distance of 4 mm to a 25 G nozzle, electrified using 6 kV and delivered using an air pressure of 3 bar. The electrified jet was deposited onto a metal collector into 48 × 96 mm rectangular samples with a 200 µm spacing fiber. To obtain circular reinforcement frames, the fiber was cut Rayjet 50C30 machine with a 30W C0_2_ laser source, 655 nm laser pointer, and 1.5 m s^−1^ engraving speed (Trotec Laser, Austria) to 9 mm circumference. The cutting process was varied through the laser speed and power percentage in the Minimanager 2.4.1 software as shown in Figure S2 (Supporting Information). A scanning electron microscope image of the scaffold morphology is shown in Figure S3 and Video S1 (Supporting Information).

### Cell Culture

HEK293 cells (CRL‐1573; ATCC – Global Biosource Center, Manassas, VA) were cultured in Minimal Essential Medium (21090‐055, Gibco, MA) supplemented with 10% Fetal Bovine Serum (10270‐106 Life Technologies, MA), 50 µg mL^−1^ penicillin/streptomycin (15140‐122 Life Technologies, MA), 1% 200 × 10^−3^ m GlutaMAX (35050‐038 Life Technologies, MA), 1% 100 × 10^−3^ m sodium pyruvate (11360‐039 Life Technologies, MA). Transfected HEK‐293 cells were used as controls in Western blot experiments and immunocytochemical staining.

### Transfection

HEK‐293 cells were transiently transfected with GlyR*α*1 using a modified calcium phosphate precipitation method and were used as positive controls for immunocytochemical staining and western blot analysis. Briefly, a mixture of plasmid DNA, CaCl_2_, and 2xHBS buffer (50 × 10^−3^ m 4‐(2‐hydroxyethyl)‐1‐piperazineethanesulfonic acid (HEPES), 12 × 10^−3^ m glucose, 10 × 10^−3^ m KCl, 280 × 10^−3^ m NaCl, 1.5 × 10^−3^ m Na_2_HPO_4_) was applied onto the cells. All experiments were performed 48 h after transfection.

### Isolation of Spinal Cord Neurons

Spinal cord neurons were prepared from wildtype CD‐1 mice at E12.5. Experiments were approved by the local veterinary authority (Veterinäramt der Stadt Würzburg, Germany) and the Ethics Committee of Animal Experiments, i.e., Regierung von Unterfranken, Würzburg, Germany (license no.: FBVVL 568/200‐324/13). Briefly, at embryonic stage E12.5 embryos were taken out of the euthanized mother mouse and transferred under a Binocular. Spinal cords were dissected out of the embryos and collected in neurobasal medium (21103‐049 Life Technologies, MA) on ice. After all spinal cords were harvested, the tissue was trypsinized using 10 mL of trypsin/EDTA (1 mg mL^−1^) and 100 µL of DNase I (final concentration, 0.1 mg mL^−1^), incubating the suspension at 37 °C for 30 min. Trypsinization was stopped with 100 µL of fetal calf serum (final concentration, 10%). After a three‐step trituration, the cells were centrifuged at 300 rpm for 15 min. Trituration was repeated. Spinal cord neurons were grown in neurobasal medium supplemented with 1% 200 × 10^−3^ m L‐Glutamine (25030‐024 Life Technologies, MA), 1% B27 (17504‐044 Life Technologies, MA), 2% horse serum (16050‐122 Gibco, MA), 0.1% 10 ng mL^−1^ brain‐derived neurotrophic factor (BDNF) and ciliary neurotrophic factor (CNTF) (AG Sendtner, Institute for Clinical Neurobiology, Würzburg, Germany) with an exchange of 50% medium after 6 days in culture. For immunocytochemical studies in a 2D environment, 150 000 spinal cord neurons were seeded in 3 cm dishes containing 4 poly‐*D*‐lysine coated coverslips; for the 3D environment 450 000 spinal cords cells per scaffold were used. Matrigel was used at a protein concentration of 4 mg mL^−1^ (734‐0271 Corning, NY). In total five independent murine spinal cord preparations were performed for 2D analysis and four preparations were carried out to conduct 3D immunocytochemical stainings.

### Membrane Preparation of Native Spinal Cord Tissue and Transfected HEK293 Cells

Spinal cord tissue at embryonic (E) stages E10, E12, E15, E17, and post‐natal (P) stages P0, P3, P7, P10, and P12 were used for protein analysis. As controls, transfected HEK‐293 cells were used. Spinal were cords dissected and immediately processed or frozen at −80 °C until use. All steps were performed on ice as previously described.^[^ ^54^ ^]^ Briefly, samples were mixed with 1–2 mL cold Buffer H (1% 250 × 10^−3^ m K_2_HPO_4_, 1% 250 × 10^−3^ m KH_2_PO_4_, 0.5% 250 × 10^−3^ m EGTA pH 8, 0.5% 250 × 10^−3^ m EDTA pH 8, 1 tablet Roche Complete EDTA free Protease Inhibitor Cocktail, dH_2_O, 0.25% 200 × 10^−3^ m phenylmethylsulfonyl fluoride (PMSF)), homogenized by a glass rod in a glass homogenisator, and further processed with an *Ultra‐Thurrax* (United Technologies Corporation (UTC)). After a second homogenization process with the *Ultra‐Thurrax*, the pellet was resuspended in Buffer B (2.1% 3 m KCl, 2.5% 250 × 10^−3^ m K_2_HPO_4_, 2.5% 250 × 10^−3^ m KH_2_PO_4_, 1 tablet Roche Complete EDTA‐free Protease Inhibitor Cocktail, dH_2_O, 0.025% DNAse). Protein concentration of each sample was determined using the Bradford assay.

### SDS‐PAGE and Western Blot

SDS‐PAGE was carried out with membrane preparations (10 µg µL^−1^). Proteins samples were separated by SDS‐Page using 11% gels followed by transfer of separated proteins onto a nitrocellulose membrane (GE Healthcare, Little Chalfont, UK). After blocking for 1 h with 5% BSA in TBS‐T (TBS with 1% Tween 20), membranes were incubated with primary antibodies over night at 4 °C (VGAT (131 003, Synaptic Systems, Göttingen, Germany; 1:2500), gephyrin (147 111 Synaptic Systems, Göttingen, Germany; 1:1000), GlyR *α*1 (HPAD 16AD/6502, Merck, Darmstadt, Germany; 1:1000)). Proteins were visualized with the help of horseradish peroxidase (111‐036‐003 and 115‐035‐146, Dianova, Hamburg, Germany) and detected through chemiluminescence with CCD cameras by clarity Western ECL Substrate (Clarity Western Peroxide Reagent, BioRad 170‐5061, CA). GAPDH (CB 1001, Merck, Darmstadt, Germany; 1:1000) was used as loading control.

### Immunocytochemical Studies

Neurons were washed with PBS, fixed with 4% PFA/sucrose for 15 min (2D) or 2% PFA/sucrose for 5 min (3D), followed by blocking with 5% goat serum for 30 min. For staining of intracellular proteins, 0.1% Triton‐X100 was added. Cells were incubated for 1 h with primary antibodies VGAT (131 308; 1:500), GlyR *α*1 (146 118, 1:500), and gephyrin (147 111, 1:500) for intracellular staining and GlyR *α*1 (146 118, 1:500) and gephyrin (147 111, 1:500) for surface staining (all Synaptic Systems, Göttingen, Germany). After three washing steps, cells were incubated with secondary antibodies (Dianova, Hamburg Germany) for 1 h (Alexa488 (115‐546‐003), Cy5 (111‐175‐006), Cy3 (111‐165‐003), and Cy3 (106 165 003 Jackson Immunoresearch, PA). 3D reinforcement frames were mounted with ProLong Antifade Mountant with NucBlue (P36983, ThermoFisher Scientific, Waltham, MA).

Spinal cord neurons were fixed at days 3, 7, 10, 14, 17, 21 in culture for 2D immunocytochemical stainings and kept at 4 °C in PBS till final staining. All immunocytochemical stainings were performed at the same day to ensure equal conditions, facilitating quantitative image analysis.

Staining of neuronal cells from 3D cultures (3–21 days in culture) were performed separately and on the day of fixation.

### Calcium Imaging

For cell calcium analysis, spinal cord neurons DIV 14 were labeled with the high affinity calcium indicator Oregon Green 488 BAPTA‐1AM (Thermo Fischer Scientific, Waltham, MA). 0.5 µL of a 5 mm stock solution in 20% Pluronic F‐127 in dimethyl sulfoxide (DMSO) was dissolved in 500 µL imaging solution for 30 min at 37 °C and 5% CO_2_. The imaging solution consisted of (in × 10^−3^ m) 119 NaCl, 4.5 KCl, 1 MgCl_2_, 2 CaCl_2_, 1.2 NaH_2_PO_4_, 26 NaHCO_3_, 10 glucose, 10 HEPES, pH7.4, adjusted with NaOH. Calcium imaging was performed under continuous perfusion with the imaging solution. Image series were captured at 10 Hz with a Rolera XR Mono fast 1394 CCD camera (Qimaging, Surrey, Canada) and StreamPix4 Software (Norpix, Montreal, Canada) under continuous illumination with a cooled epifluorescent light source for 470 mm (Visitron Systems, Puchheim, Germany) and analyzed by ImageJ.

### Confocal Microscopy and Image Acquisition

Images were acquired using an inverted Olympus iX81 microscope equipped with diode lasers of 405 nm (DAPI), 473 nm (Alexa488), 559 nm (Cy3), and 635 nm (Cy5). All images shown were acquired using an Olympus UPLSAPO 60x (oil, numerical aperture: 1.35) objective. For 3D PCL frames, stacks of 1 µm thickness were acquired. In total 5 images were acquired for each 2D sample and 3 images for each 3D sample. Areas of low neuron density were chosen to improve counting of molecules and colocalizations later using the ImageJ software.

### Processing of Images

ImageJ (1.52)/Fiji was used to trace dendrites with the ImageJ tool NeuronJ.^[^ ^55^ ^]^ Synaptic proteins and colocalizations were counted using the tool SynapCountJ (unit: density/100 µm dendrite) with a pixel diameter of 10. This tool is configured to count colocalizations of two overlayed images.

For 2D and 3D image analyses, protein densities of 10–20 dendrites each were evaluated in total using 2–5 images from every sample. For all images gephyrin as the scaffolding protein of glycinergic synapses was used to trace the dendrites. For all images a specific range was set, to clearly mark pixels resembling synaptic proteins in the image. To avoid bias, the thresholds were kept in a certain range for samples that were stained at the same day. Each count was afterward double checked by eye. For the counting of single proteins, the threshold was also set to a value where all clearly distinguishable dots were included in the counting.

When determining the colocalization density, two different images (e.g., gephyrin and glycine receptor channel) were loaded into ImageJ. The exact same settings used for counting of the proteins in the single channels were assumed without exception.

To avoid additional bias, blind analysis of images acquired for one individual experiment was performed. Furthermore, image acquisition at the Olympus microscope and image analysis using ImageJ were carried out by two different persons. Imaris was used for 3D reconstruction (Oxford Instruments, Abingdon, UK).

### Mechanical Analysis

For mechanical analyses of the samples, we used the Discovery HR‐3 Rheometer from TA Instruments (TA Instruments, Inc., Newcastle, USA). To avoid measurement uncertainties, the height of each sample was determined before testing using an inverse microscope Axio Observer 7 (Carl Zeiss AG, Oberkochen, Germany). The average thickness of the samples was 141 ± 3 µm for the MEW‐frames and 642 ± 109 µm for pure Matrigel, the MEW‐frame‐Matrigel samples and the 3D primary spinal cord cultures including the MEW‐frame matrix composite. The sample diameter was identical to the MEW‐frame diameter with *d* = 9 mm for all samples. As the samples were kept hydrated with neurobasal medium during testing with the assumption that they slide along the specimen holders’ surfaces, yielding a homogeneous deformation throughout the sample. To mimic in vivo conditions, all tests were performed at a constant temperature of 37 °C. The testing protocol started with a cyclic compression test with three loading cycles, a maximum strain of 15% and a velocity of 40 µm s^−1^. A stress relaxation test was conducted at the same maximum strain and velocity and a holding time of 300 s.

### Statistical Analyses

Statistical analysis for mechanical testing of MEW‐frame, pure Matrigel, and MEW‐frame‐Matrigel samples was performed using students *t‐*test and mechanical testing of 3D primary spinal cord cultures was performed using one‐way ANOVA with Tukey–Kramer post‐test using Statistics Toolbox in MATLAB version R2019a 9.6.0.1072779 (MathWorks, USA). A *p*‐value lower than 0.05 was considered to be significant.

Statistical analysis of immunocytochemical intensities and colocalizations was performed using one‐way ANOVA with Dunnett's post‐test and students *t‐*test using GraphPad Prism version 9.0.2(161) for Windows (GraphPad Software, La Jolla California USA). A *p*‐value lower than 0.05 was considered to be significant.

## Conflict of Interest

The authors declare no conflict of interest.

## Author Contributions

C.V., N.S., S.B., P.D. participated in research design. E.B., N.F., N.S., and J.F. conducted the experiments. N.F.; N.S., J.F., S.B. performed the data analysis. N.F. and S.B. contributed to the initial draft. C.V., N.S., and P.D. wrote the manuscript. All authors contributed to the article and approved the submitted version.

## Supporting information

Supporting Information

Supplemental Video 1

Supplemental Video 2

## Data Availability

The data that supports the findings of this study are available in the supplementary material of this article.
