# Supplementary material for: Spinal Cord Neuronal Network Formation in a 3D Printed Reinforced Matrix—A Model System to Study Disease Mechanisms
Source: Adv Healthc Mater. 2021 Aug 5;10(19):2100830. doi: 10.1002/adhm.202100830 (PMC11469053; doi:10.1002/adhm.202100830)
Supplement: Supplementary file 1 — Supporting Information [file ADHM-10-2100830-s001.pdf]

© 2021 Wiley-VCH GmbH

ADVANCED HEALTHCARE MATERIALS

Supporting Information

for *Adv. Healthcare Mater.*, DOI: 10.1002/adhm.202100830

# Spinal cord neuronal network formation in a 3D printed reinforced matrix – a model system to study disease mechanisms

*Natalie Fischhaber, Jessica Faber, Ezgi Bakirci, Paul D. Dalton, Silvia Budday, Carmen Villmann, Natascha Schaefer**

Copyright WILEY-VCH Verlag GmbH & Co. KGaA, 69469 Weinheim, Germany, 2018.

# Supporting Information

## Spinal cord neuronal network formation in a 3D printed reinforced matrix – a model system to study disease mechanisms

Natalie Fischhaber, Jessica Faber, Ezgi Bakirci, Paul D. Dalton, Silvia Budday, Carmen Villmann, Natascha Schaefer*

**Figure S1:** Schematical overview of an inhibitory synapse

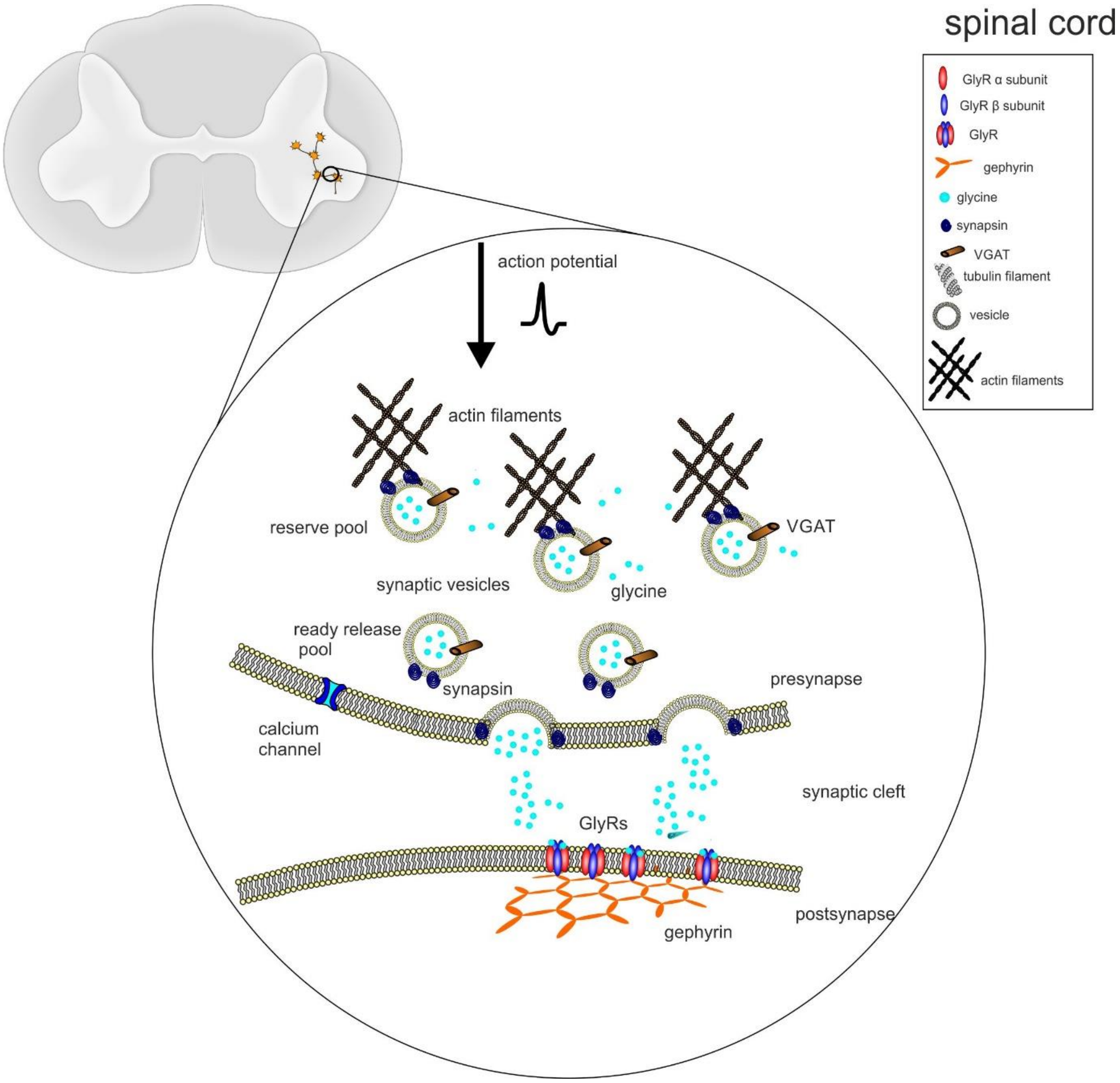


*top* - spinal cord depicted with an enlarged view of the inhibitory synapse (circle). Pre- and postsynapse are shown by opposing membranes. Typical structural proteins for inhibitory synapse formation that are monitored in the present study are labelled: synapsin – localized at synaptic vesicles. Synaptic vesicles contain chemical neurotransmitters: e.g. glycine. Glycine is loaded into synaptic vesicles by the vesicular-GABA transporter (VGAT). At the postsynapse, ligand-gated glycine receptors (GlyR) are anchored to the post-synaptic membrane via the scaffold protein gephyrin.

**Figure S2:** Laser Cutting of PCL reinforcement frames. The laser power was set to 2.1W on the software Minimanager 2.4.1 and the cutting was varied through the laser speed percentage and number of passes in the same software.  Stereomicroscopy of samples in this study that were laser cut with A-B) 6 cm.s$^{-1}$, C) 4.5 cm.s$^{-1}$ and D-E) 3 cm.s$^{-1}$ indicate only partially laser cut samples, when the cutting speed was too rapid or overly melted edges when the speed was too low. F) shows the ideal cutting outcome with a more rapid speed of 6 cm.s$^{-1}$ but with four circular passes to produce the scaffolds used in this study.

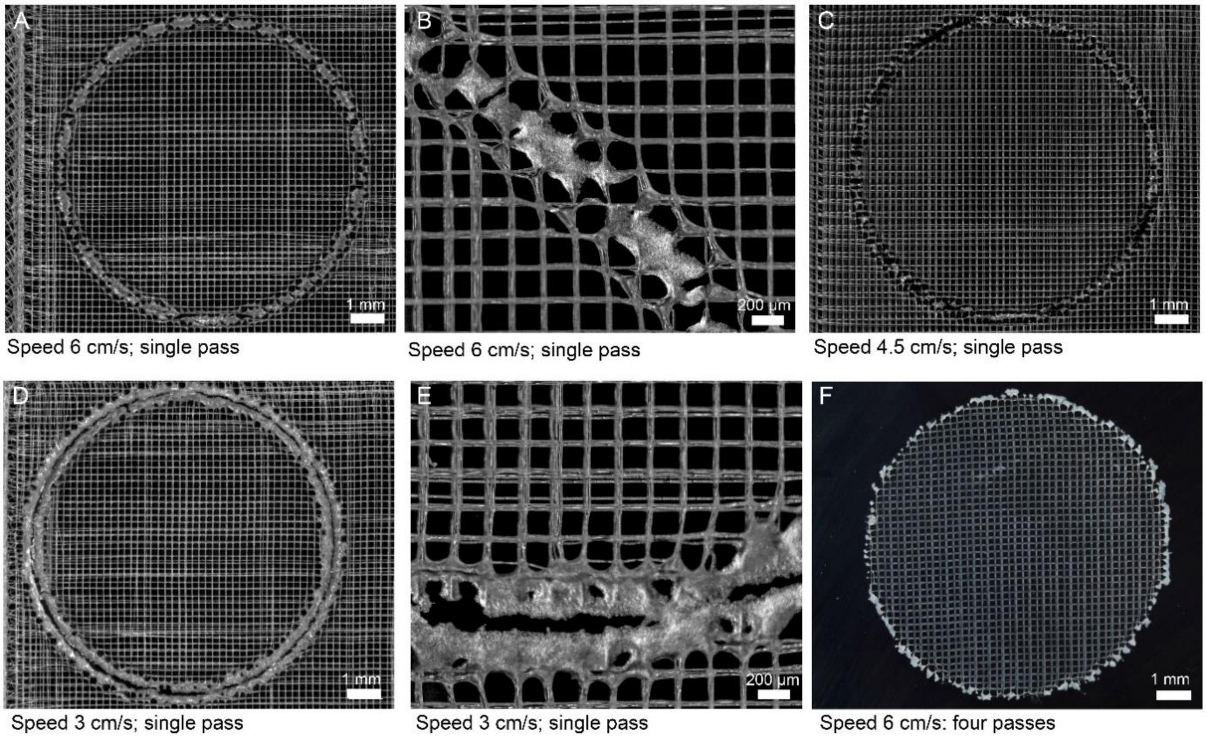

**Figure S3:** Scanning electron microscopy image of the melt electrowritten PCL reinforcing frames used in this study.

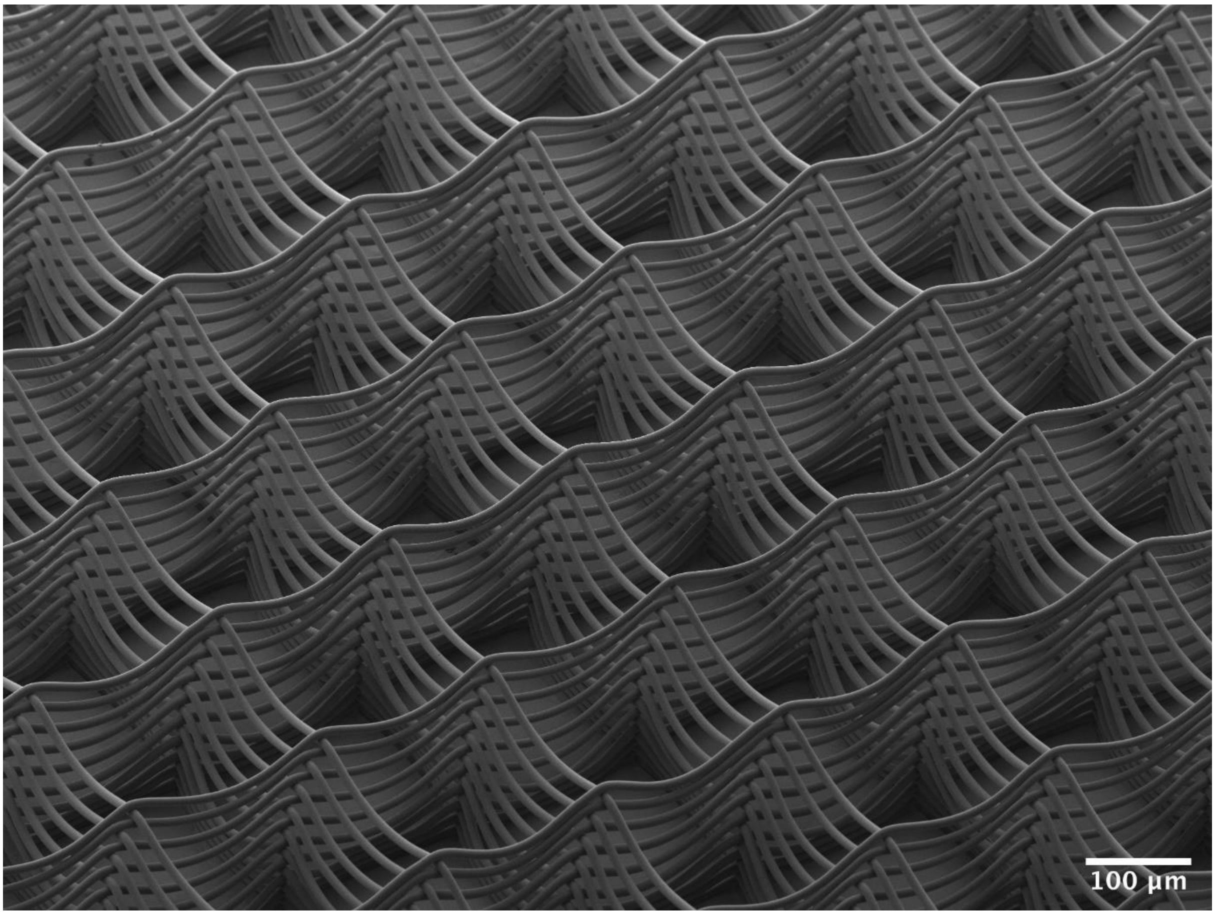

**Table S1:** Cyclic compression tests of MEW-frame, Matrigel and MEW-frame + Matrigel

| | | MEW-frame | Matrigel | MEW-frame + Matrigel |
|---|---|---|---|---|
| | n | 9 | 5 | 11 |
| **Maximum values of Cyclic Compression tests** | 1st cycle | -1.373 ± 0.086 | -0.4209 ± 0.119 | -0.5796 ± 0.315 |
| | 2nd cycle | -1.3492 ± 0.064 | -0.2159 ± 0.045 | -0.3824 ± 0.151 |
| | 3rd cycle | -1.3484 ± 0.044 | -0.2249 ± 0.051 | -0.3091 ± 0.106 |
| 1st cycle vs 2nd cycle | p | 0.8929 ns | 0.0319 * | 0.0655 * |
| 1st cycle vs 3rd cycle | p | 0.9284 ns | 0.0394 * | 0.0106 * |
| x vs MEW-frame | 1st cycle p | - | <0.0000 **** | <0.0000 **** |
| | 2nd cycle p | - | <0.0000 **** | <0.0000 **** |
| | 3rd cycle p | - | <0.0000 **** | <0.0000 **** |

Significance values: *p<0.05; ****p<0.0001, ns = not significant; n = number of measured constructs.

**Table S2:** Whole cell protein amount of VGAT, gephyrin and GlyR

| | | DIV3 | DIV7 | DIV10 | DIV14 | DIV17 | DIV21 |
|---|---|---|---|---|---|---|---|
| **Whole cell** | 2D n | 100 | 100 | 81 | 100 | 80 | 79 |
| | 3D n | 17 | 30 | 39 | 40 | 40 | 40 |
| **VGAT** | 2D mean ± SEM | 53.2±3.1 | 60±2 | 47±2.1 | 46.7±2.2 | 45.2±2.1 | 47.3±2.1 |
| | 3D mean ± SEM | 47.1±4.1 | 45.9±4.9 | 48±3.7 | 48.2±3.7 | 49.2±3.8 | 53.8±3.7 |
| 2D vs 3D | p | 0.4322 ns | 0.0037 ** | 0.7764 ns | 0.7237 ns | 0.3159 ns | 0.1031 ns |
| DIVx vs DIV21 | 2D p | 0.2670 ns | 0.0021 ** | 0.9998 ns | <0.9997 ns | 0.9585 ns | - |
| | 3D p | 0.7809 ns | 0.4386 ns | 0.6916 ns | 0.7224 ns | 0.8488 ns | - |
| **Whole cell** | 2D n | 180 | 200 | 181 | 200 | 160 | 158 |
| | 3D n | 47 | 50 | 79 | 80 | 80 | 80 |
| **gephyrin** | 2D mean ± SEM | 63.5±2.1 | 65.6±1.4 | 62.8±1.3 | 62±1.6 | 60.6±1.5 | 63.6±1.8 |
| | 3D mean ± SEM | 39.5±3.7 | 56±4.2 | 57±2.4 | 58.3±2.5 | 55.6±3.2 | 63.2±2.5 |
| 2D vs 3D | p | <0.0001 **** | 0.0057 ** | 0.0217 * | 0.2070 ns | 0.1111 ns | 0.9137 ns |
| DIVx vs DIV21 | 2D p | >0.9999 ns | 0.8228 ns | 0.9981 ns | 0.9457 ns | 0.6023 ns | - |
| | 3D p | <0.0001 **** | 0.3594 ns | 0.3743 ns | 0.6142 ns | 0.1942 ns | - |
| **Whole cell** | 2D n | 100 | 100 | 81 | 100 | 80 | 80 |
| | 3D n | 17 | 30 | 39 | 40 | 40 | 40 |
| **GlyR** | 2D mean ± SEM | 42.17±3.4 | 60±3.1 | 49±3 | 39.6±2.6 | 61.2±4.1 | 69.2±3.8 |
| | 3D mean ± SEM | 4.2±1.3 | 25.5±5.5 | 50±4 | 55.4±3.6 | 55.2±2.9 | 52.8±4.1 |
| 2D vs 3D | p | <0.0001 **** | <0.0000 **** | 0.8310 | 0.0011 ** | 0.3272 | 0.0080 ** |
| DIVx vs DIV21 | 2D p | <0.0001 **** | 0.1580 ns | 0.0003 *** | <0.0001 **** | 0.3462 ns | - |
| | 3D p | <0.0001 **** | <0.0001 **** | 0.9810 ns | 0.9857 ns | 0.9905 ns | - |

Significance values: *p<0.05; **p<0.01, ***p<0.001; ****p<0.0001, ns = not significant; n = number of counted dendrites

**Table S3:** Intracellular colocalization of VGAT vers GlyR and gephyrin versus GlyR

| | | | DIV3 | DIV7 | DIV10 | DIV14 | DIV17 | DIV21 |
|---|---|---|---|---|---|---|---|---|
| **Whole Cell Co-localization** | 2D | n | 100 | 30 | 39 | 100 | 80 | 80 |
| | 3D | n | 17 | 30 | 39 | 40 | 40 | 40 |
| VGAT vs GlyR | 2D mean ± SEM | | 14.24±1.9 | 28.9±2.2 | 24.5±2.3 | 20.2±1.9 | 31.7±2.8 | 36.3±2.5 |
| | 3D mean ± SEM | | 1.3±0.7 | 16.1±3.9 | 29.6±2.8 | 32.6±3.8 | 31.9±2.7 | 35.1±3.1 |
| 2D vs 3D | | p | <0.0069 ** | 0.0056 ** | 0.1902 ns | 0.0016 ns | 0.9625 ns | 0.7741 ns |
| DIVx vs DIV21 | 2D | p | <0.0001 **** | 0.0882 ns | 0.0025 ** | <0.0001 **** | 0.5129 ns | - |
| | 3D | p | <0.0001 **** | 0.003 *** | 0.6011 ns | 0.9728 ns | 0.9275 ns | - |
| **Whole cell Co-localization** | 2D | n | 100 | 100 | 81 | 100 | 80 | 80 |
| | 3D | n | 17 | 30 | 39 | 40 | 40 | 40 |
| gephyrin vs GlyR | 2D mean ± SEM | | 22.2±2.3 | 31.6±2.4 | 28.6±2.1 | 24.6±1.9 | 30.9±2.9 | 35.3±2.7 |
| | 3D mean ± SEM | | 0.25±0.2 | 15±3.2 | 31.6±3.3 | 39.3±3.3 | 35.7±3.5 | 37.9±4.1 |
| 2D vs 3D | | p | <0.0001 **** | 0.0006 *** | 0.4328 ns | <0.0001 **** | 0.3226 ns | 0.5959 ns |
| DIVx vs DIV21 | 2D | p | 0.0005 *** | 0.6875 ns | 0.1948 ns | 0.0066 ** | 0.5885 ns | - |
| | 3D | p | <0.0001 **** | <0.0001 **** | 0.5515 ns | 0.9981 ns | 0.9898 ns | - |

Significance values: **p<0.01, ***p<0.001; ****p<0.0001, ns = not significant; n = number of counted dendrites.

**Table S4:** Surface protein amount and amount of surface colocalization of GlyR

| | | | DIV3 | DIV7 | DIV10 | DIV14 | DIV17 | DIV21 |
|---|---|---|---|---|---|---|---|---|
| **Surface GlyR** | 2D | n | 80 | 100 | 100 | 100 | 80 | 78 |
| | 3D | n | 30 | 30 | 40 | 40 | 40 | 40 |
| | 2D mean ± SEM | | 14.5±2.4 | 18.6±2.3 | 24.1±1.9 | 39.3±2.8 | 56.2±3.3 | 50.7±2.9 |
| | 3D mean ± SEM | | 14±3.5 | 14±3.5 | 43.1±4.1 | 60.8±3.2 | 61.7±3.2 | 62.3±3.8 |
| 2D vs 3D | | p | 0.9148 ns | 0.3089 ns | <0.0001 **** | <0.0001 **** | 0.2857 ns | 0.0207 * |
| DIVx vs DIV21 | 2D | p | <0.0001 **** | <0.0001 **** | <0.0001 **** | 0.0092 ** | 0.482 ns | - |
| | 3D | p | <0.0001 **** | <0.0001 **** | 0.0005 *** | 0.9978 ns | 0.9998 ns | - |
| **Surface Co-localization** | 2D | n | 80 | 100 | 99 | 100 | 80 | 78 |
| | 3D | n | 30 | 20 | 40 | 40 | 40 | 40 |
| gephyrin vs GlyR | 2D mean ± SEM | | 7.6±1.5 | 9.7±1.3 | 10.5±1.1 | 23.8±2.1 | 34.8±2.5 | 29.5±2.4 |
| | 3D mean ± SEM | | 8.3±2.8 | 17.6±2.5 | 28.4±3.2 | 45.2±3.2 | 45.2±4.6 | 52.5±3.9 |
| 2D vs 3D | | p | 0,8043 ns | 0.0125 * | <0.0001 **** | <0.0001 **** | 0.0324 * | <0.0001 **** |
| DIVx vs DIV21 | 2D | P | <0.0001 **** | p<0.0001 **** | <0.0001 **** | 0.1149 ns | 0.1867 ns | - |
| | 3D | p | <0.0001 **** | <0.0001 **** | <0.0001 **** | 0.4497 ns | 0.4548 ns | - |

Significance values: *p<0.05; **p<0.01, ***p<0.001; ****p<0.0001, ns = not significant; n = number of counted dendrites.

**Table S5:** Cyclic compression tests of spinal cord-MEW-frame-Matrigel constructs at DIVs 0, 3, 7, 10, and 14

| | | DIV0 | DIV3 | DIV7 | DIV10 | DIV14 |
|---|---|---|---|---|---|---|
| **Maximum values of Cyclic Compression tests** | n | 10 | 10 | 10 | 10 | 10 |
| | 1st cycle | -0.6790 ± 0.236 | -0.7679 ± 0.3 | -1.039 ± 0.281 | -0.8318 ± 0.291 | -0.8328 ± 0.136 |
| DIVx vs DIV0 | p | - - | 0.9384 ns | 0.0258 * | 0.6801 ns | 0.6967 ns |

Significance values: *p<0.05; ns = not significant; n = number of measured constructs.

**Supporting Video 1** Caption: High resolution SEM image of the PCL MEW reinforcement frames, with 9.05 ± 0.2µm diameter individual fibers and 200µm spacing in a grid pattern. Distortions at the edges of the scaffold at low magnification are due to electron microscope aberrations.

**Supporting Video 2** Caption: 3D reconstruction of immunocytochemical z-stack of a DIV14 composite shown in **Figure 7a.** Proteins marked are VGAT (cyan), GlyR(magenta), Nuclei (DAPI), MEW-scaffold shows an autofluorescence in the DAPI-channel.
